# Valsalva sinus perforation caused by Cor-Knot during totally endoscopic minimally invasive aortic valve replacement

**DOI:** 10.1016/j.xjtc.2025.06.020

**Published:** 2025-06-28

**Authors:** Akitoshi Yamada, Chihiro Okubo, Ryo Tohma, Hidekazu Nakai, Yoshihisa Morimoto, Kunio Gan, Tatsuro Asada

**Affiliations:** The Department of Cardiovascular Surgery, Kita-Harima Medical Center, Ono, Hyogo, Japan

**Figure:**
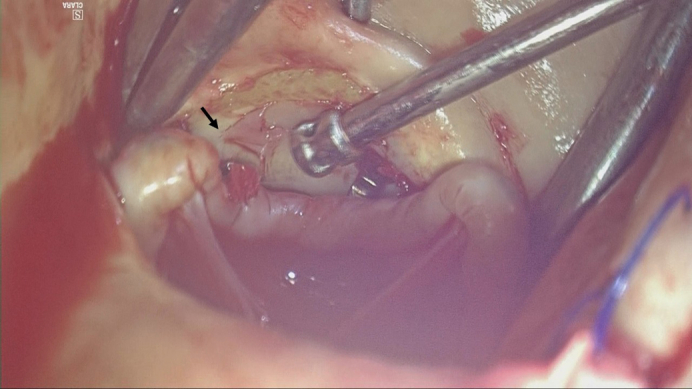
A Valsalva sinus perforation matching the Cor-Knot fastener width was identified.

**Central Message**

In endoscopic minimally invasive cardiac surgery aortic valve replacement, special caution is required when using the Cor-Knot in small Valsalva sinuses.

The Cor-Knot (LSI Solutions) is widely used to facilitate suture ligation in heart valve surgery. While its safety and efficacy are well established,^1^ isolated cases of prosthetic valve injury during aortic valve replacement (AVR) have been reported, possibly related to mechanical interaction between the fastener and the prosthetic tissue.^2^^,^^3^ We report a rare case of Valsalva sinus perforation attributed to Cor-Knot use during totally endoscopic minimally invasive cardiac surgery (MICS) AVR.

## Case Description

A 75-year-old woman (height, 154 cm; weight, 37 kg; body mass index, 15.6 kg/m^2^) receiving chronic corticosteroid therapy for Sjögren's syndrome and systemic lupus erythematosus presented with symptomatic severe aortic stenosis. She underwent totally endoscopic MICS AVR via a 2-window approach,^4^ using 2 separate ports in the fourth intercostal space, one as a camera port and the other as the main working window (4 cm), and a 2-cm auxiliary window in the second intercostal space for left-hand instrumentation.

The patient exhibited a small aortic annulus measuring 20 mm and a narrow left ventricular outflow tract measuring 18 mm. Antegrade selective cardioplegia was successfully administered, and the heavily calcified aortic valve was excised under optimal endoscopic visualization. A 19-mm Epic bioprosthetic valve (Abbott Vascular) was implanted in the supra-annular position (Video 1).

Cor-Knot was used to secure the annular sutures, orienting the fasteners outward according to the manufacturer's guidelines to avoid leaflet impingement. However, immediately following aortic cross-clamp removal, significant oozing was observed from the anterior aortic root, necessitating emergent conversion to full sternotomy. Hemostasis proved challenging under beating-heart conditions, requiring reapplication of the cross-clamp, and the heart was arrested.

**Figure 1:**
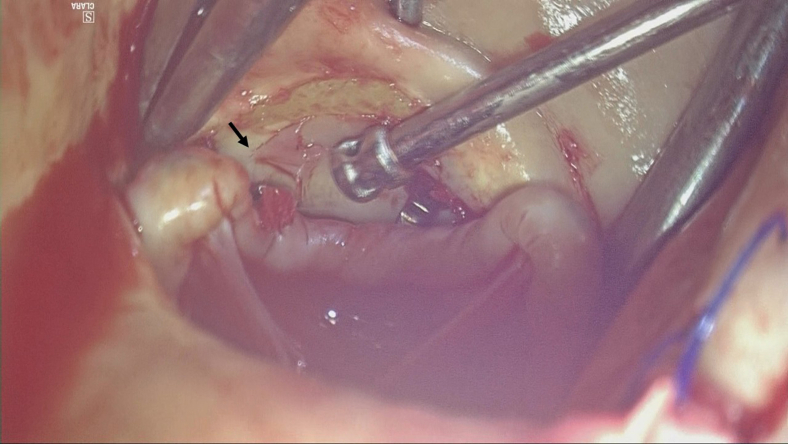
Still image from Video 1. A solitary perforation was observed on the Valsalva sinus, directly caused by mechanical injury from the Cor-Knot fastener. The *black arrow* indicates the Valsalva sinus perforation corresponding to the width of the Cor-Knot fastener.

Inspection revealed a 3-mm perforation at the Valsalva sinus near the right coronary cusp, adjacent to the commissure between the right and left coronary cusps (Figure 1). The perforation was repaired from both the luminal (internal) and adventitial (external) sides of the aorta with 1 mattress suture each, using 5-0 polypropylene and polytetrafluoroethylene pledgets. The patient's postoperative course was uneventful, and she was discharged without major complications.

## Discussion

The Cor-Knot system provides clear advantages in MICS by improving the consistency and efficiency of suture fixation. Nevertheless, rare but serious complications involving aortic root injury have been documented.^5^ In the present case, multiple mechanisms may explain the Valsalva sinus perforation. Although tissue fragility associated with advanced age, female sex, and chronic immunosuppression due to long-term steroid use may have contributed to the risk of structural injury in this case, the following mechanisms are also considered possible.

First, direct trauma from the Cor-Knot shaft during deployment may have injured the fragile Valsalva wall. In totally endoscopic MICS, the Cor-Knot shaft is inserted through the main port in the fourth intercostal space and approaches the valve at a rightward oblique angle, limiting maneuverability. Second, although outward fastener orientation is recommended to prevent leaflet injury, this may paradoxically predispose small aortic roots—those requiring the smallest available bioprosthesis—to Valsalva damage. The rigid metal edge of an outward-tilted fastener can directly impinge on adjacent fragile tissue. Third, the injury might have occurred either during firing owing to localized mechanical stress or progressively after declamping under physiologic pulsatile motion.

**Figure 2:**
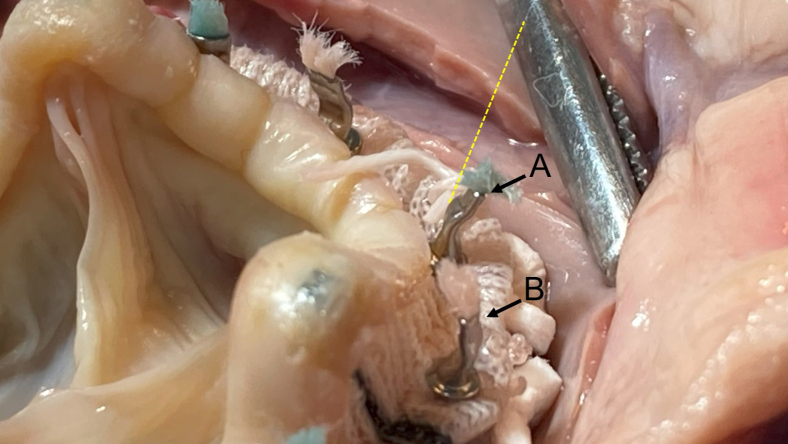
Ex vivo porcine heart simulation demonstrating Cor-Knot deployment during Epic valve implantation. A, Recommended orientation with the fastener edge facing the Valsalva sinus. B, Reverse orientation with the edge directed away. The *yellow dotted line* represents the endoscopic shaft trajectory. The *black arrow* indicates the Valsalva sinus perforation corresponding to the width of the Cor-Knot fastener.

To investigate this hypothesis, we conducted an ex vivo simulation using porcine hearts. In all 3 repeated experiments under totally endoscopic conditions, the Cor-Knot fastener edge consistently pointed perpendicularly toward the right coronary cusp Valsalva wall, suggesting an elevated risk of localized wall injury with standard fastener orientation (Figure 2, *A*). While the Epic valve's protective leaflet design may support the theoretical benefit of inward fastener orientation in reducing Valsalva wall stress, this technique remains unvalidated in vivo and warrants further investigation before clinical use (Figure 2, *B*). We also advocate strict intraoperative endoscopic visualization during Cor-Knot firing and recommend gentle, deliberate deployment. Avoiding sudden or forceful activation may further mitigate tissue injury.

This case highlights a rare but significant complication associated with Cor-Knot use during totally endoscopic MICS AVR. Surgeons should recognize the potential for Valsalva injury, particularly in small aortic roots. Adjustment of fastener orientation, precise shaft alignment, and careful visualization during deployment are crucial for procedural safety. Specifically, inward orientation of Cor-Knot fasteners may offer an additional layer of protection when implanting Epic valves in patients with small aortic annulus. In addition to careful Cor-Knot handling, alternatives such as sutureless valves, direct-vision AVR, or transcatheter AVR may be considered in small annulus or fragile tissue cases, as seen here.

## Conflict of Interest Statement

The authors reported no conflicts of interest.

The *Journal* policy requires editors and reviewers to disclose conflicts of interest and to decline handling or reviewing manuscripts for which they may have a conflict of interest. The editors and reviewers of this article have no conflicts of interest.

## Supplementary Data

Video 1 The prosthetic valve being secured with a Cor-Knot fastener in a small Valsalva sinus. Video available at: https://www.jtcvs.org/article/S2666-2507(25)00269-X/fulltext.
