# Supplementary figures and images for: Valsalva sinus perforation caused by Cor-Knot during totally endoscopic minimally invasive aortic valve replacement
Source: JTCVS Tech. 2025 Jun 28;33:72–4. doi: 10.1016/j.xjtc.2025.06.020 (PMC12529675; doi:10.1016/j.xjtc.2025.06.020)

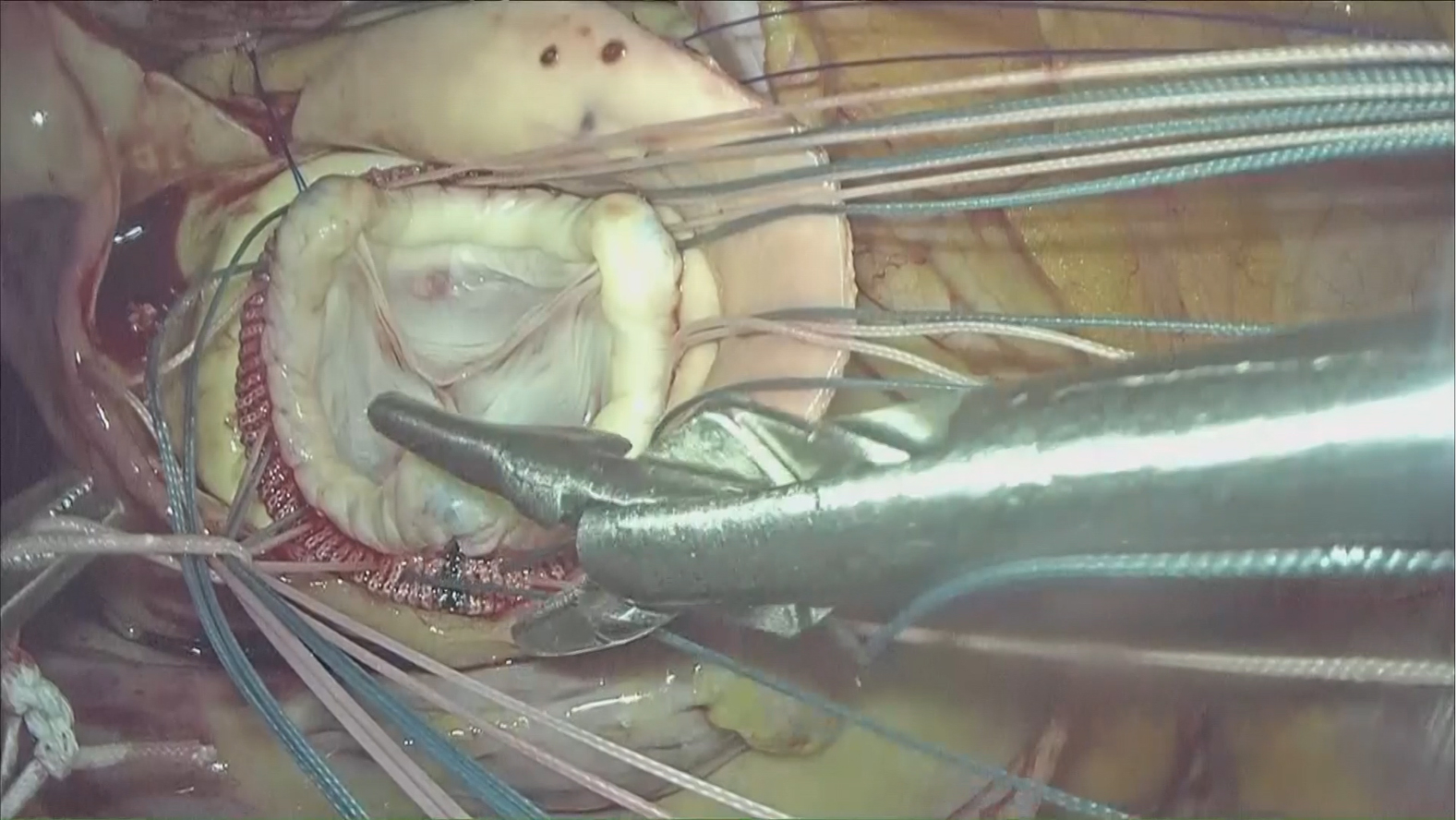

Supplement: Video 1 — The prosthetic valve being secured with a Cor-Knot fastener in a small Valsalva sinus. Video available at: https://www.jtcvs.org/article/S2666-2507(25)00269-X/fulltext. [file fx2.jpg]
